# Unawareness of health insurance expiration status among women of reproductive age in Northern Ghana: implications for achieving universal health coverage

**DOI:** 10.1186/s41043-019-0190-4

**Published:** 2019-11-27

**Authors:** Edmund Wedam Kanmiki, Ayaga A. Bawah, James Akazili, Isaiah Agorinyah, John Koku Awoonor-Williams, James F. Phillips, Kassem M. Kassak

**Affiliations:** 1 Regional Institute for Population Studies, University of Ghana, Accra, Ghana; 2 Research and Development Division, Ghana Health Service, Accra, Ghana; 3 Swiss Tropical and Public Health Institute, University of Basel, Basel, Switzerland; 4 Policy, Planning, Monitoring and Evaluation Division, Ghana Health Service, Accra, Ghana; 5 Department of Population and Family Health, Mailman School of Public Health, Columbia University, New York City, USA; 6 Department of Health Management and Policy, Faculty of Health Sciences, American University of Beirut, Beirut, Lebanon

**Keywords:** Universal health coverage, Health insurance, Unawareness, Ghana

## Abstract

**Background:**

Ghana implemented a national health insurance scheme in 2005 to promote the provision of accessible, affordable, and equitable healthcare by eliminating service user fees. Termed the National Health Insurance Scheme (NHIS), its active enrollment has remained low despite a decade of program implementation. This study assesses factors explaining this problem by examining the correlates of insurance status unawareness among women of reproductive age.

**Methods:**

In 2015, a random probability cross-sectional survey of 5914 reproductive-aged women was compiled in the Upper East Region, an impoverished and remote region in Northern Ghana. During the survey, two questions related to the NHIS were asked: “Have you ever registered with the NHIS?” and “Do you currently have a valid NHIS card?” If the answer to the second question was yes, the respondents were requested to show their insurance card, thereby enabling interviewers to determine if the NHIS requirement of annual renewal had been met. Results are based on the tabulation of the prevalence of unawareness status, tests of bivariate associations, and multivariate estimation of regression adjusted effects.

**Results:**

Of the 5914 respondents, 3614 (61.1%) who reported that they were actively enrolled in the NHIS could produce their insurance cards upon request. Of these respondents, 1243 (34.4%) had expired cards. Factors that significantly predicted unawareness of card expiration were occupation, district of residence, and socio-economic status. Relative to other occupational categories, farmers were the most likely to be unaware of their card invalidity. Respondents residing in three of the study districts were less aware of their insurance card validity than the other four study districts. Unawareness was observed to increase monotonically with relative poverty.

**Conclusion:**

Unawareness of insurance care validity status contributes to low active enrollment in Ghana’s NHIS. Educational messages aimed at improving health insurance coverage should include the promotion of annual renewal and also should focus on the information needs of farmers and low socio-economic groups.

## Background

Ensuring access to affordable and equitable health care has become a global priority. This resulted in the 2005 World Health Assembly resolution committing member countries to ensure the financial protection of all citizens against the cost of unforeseen ill health and to plan a transition towards universal health coverage [1–3]. The United Nations estimates that nearly 150 million people face catastrophic healthcare payments annually while 100 million people transition into poverty due to direct out-of-pocket health payments [3]. In 2012, the Director-General of the World Health Organization declared in her address to the 65th World Health Assembly that universal health coverage (UHC) is “the single most powerful concept that public health has to offer” [4]. This concept encompasses three major dimensions as described by the World Health Report of 2010; the proportion of the population covered by geographically accessible primary health care, the range of health services covered (benefit package) and the proportion of health costs covered [3]. Basically, the concept of UHC seeks to ensure that all people have access to health care services when they need it, without incurring financial hardship [3].

In low- and middle-income countries, health insurance is increasingly recognized as a pillar of ensuring health care equity. Through the pooling of risks and resources, it has the potential to ensure improved access and provide risk protection against the cost of unforeseen health care expenditure [5–7].

Ghana is one of the few countries in sub-Saharan Africa that took an early lead and introduced a nationwide health insurance program [8–10]. Before independence, healthcare services in Ghana were financed solely through out-of-pocket payments [11] but this constrained access to only a few privileged in society. After independence in 1957, Ghana chose a socialist path to financing healthcare; healthcare in all public facilities was funded from general tax revenues and offered free of charge at the point of care [12]. However, in the 1970s and 1980s, economic recession was exacerbated by global macro-economic policies that severely affected government revenue resources for all health and social programs. At that time Ghana and most African countries suffered from macro-economic stagnation and indebtedness, and levels of financing required for socialist health policies were unsustainable. Nominal user fees were therefore introduced in the 1970s. But, by the mid-1980s, loan conditionality imposed by the World Bank and the IMF under the structural adjustment programs (SAP) led to substantial increases in the imposition of point of care user fees, known in Ghana as “cash and carry” [13].

Although exemptions were introduced under the “cash and carry” system for the poor, the aged and pregnant women, these were poorly implemented and under-financed [14]. The impact of this user fees however resulted in more than a two-thirds drop in health facility utilization, which was mainly among vulnerable groups [15, 16]. To forestall this, the Ministry of Health began to explore the feasibility of health insurance [2]. Trials of community-based health insurance programs emerged, initially as a pilot at Nkoranza by the St. Theresa Catholic Mission Hospital in 1992. The success of the small-scale trials led the Ghana Ministry of Health (MOH) to establish a unit for health insurance with a focus to generate evidence on the feasibility of a national social health insurance program. The unit piloted programs in the Eastern region and other parts of the country and by 2002 over 159 mutual health organizations were established [17]. In spite of this expansion, coverage remained at only about 1% of the population of Ghana at the time [17] In 2005, arising from an election campaign promise and the abundant evidence of inequalities associated with user fees, a mandatory NHIS was introduced by the then government to replace the “cash and carry” systems with the ultimate goal of achieving universal health coverage.

The Ghana National Health Insurance Scheme (NHIS) was created by an act of parliament in 2003 (Act 650), subsequently revised by Act 852 in 2012, and implemented as a national program in 2005 [18]. The NHIS is a healthcare financing strategy that aims to remove financial barriers to health care and protect all Ghanaian citizens and residents from catastrophic health expenditures which arise from user fees and other direct payments at the point of service [18–20]. It is based on a contributory model whereby service benefits are restricted to contributors. Although enrollment into the NHIS is legally mandatory, enforcement of this requirement is constrained by the fact that social policy for most of the population is governed by tradition and social norms in the informal sector rather than institutions of government. Therefore, the scheme depends on voluntary subscription with the exception of formal sector workers [21].

Aside from the payment of premiums, the NHIS is financed through a 2.5% national health insurance levy as a component of value-added tax (VAT) which is collected on selected goods and services. There is an additional 2.5% deduction of formal sector worker’s contribution to the Social Security and National Insurance Trust (SSNIT) fund. People employed in the formal sector contribute to SSNIT. Other sources of funding to the NHIS are government of Ghana budget allocations, grants, donations, and proceeds of investments made by the national health insurance council [2, 22]. Membership of the insurance scheme by the informal sector is through premium contributions. Persons below 18 or above 70 years of age, SSNIT pensioners, pregnant women, or persons deemed indigent are exempt from premium payments [21, 22].

The NHIS benefit package is a very generous package that includes outpatient and inpatient services, essential drugs, inpatient accommodation, and maternity care including cesarean delivery, dental care, eye care, and emergency care among others. Essentially about 95% of diseases in Ghana are covered by the NHIS [10].

Over the years, the scheme has proven to be successful in improving access to health care through the increase in health facility utilization, improvement in health-seeking behavior and a significant reduction in the level of out-of-pocket health care payments in the country [20, 22–24]. It has also been praised as Ghana’s ultimate strategy for achieving universal health coverage. However, over a decade of its implementation, the NHIS is still faced with major obstacles that challenge its success and sustainability. One such obstacle that threatens the operations and sustainability of the NHIS is low active membership [25]. Under the national health insurance policy, Ghana’s government objective was to ensure equitable, quality, accessible, and efficient health services to at least 60% of all Ghanaians by the year 2015 [26]. However, active coverage of the NHIS is still as low at 37% even though 63% of Ghana’s population is registered with the NHIS [27].

Previous studies that have examined the causes, determinants, and reasons for low coverage have argued that people with low socio-economic status are less likely to enroll on the NHIS due to the annual premium payments [2, 28, 29]. Other factors that have been found to be associated with enrollment are educational attainment of household heads, employment type, gender, family composition, and marital status among others [2, 22, 28, 29].

In exploring the issues regarding enrollment and active membership of the NHIS, there exists a dearth of knowledge between the two among a section of the Ghanaian populace. While enrollment is a one-time activity, active membership is subject to annual renewal through the payment of premiums and processing fee in the case of informal sector workers and only processing fee in the case of formal sector workers, without this annual renewal activity, one’s ability to access health care under the NHIS ceases with the expiration of one’s NHIS membership card. Many only get to know that their insurance has expired at the point of service when they are in need of health care; this often results in an inability to receive care under the NHIS with its resultant traumatic experience and sometimes fatal consequences. No study has examined the possibility that low active membership could be due to some subscribers’ unawareness of their membership status. This calls for an exploration of factors that may influence active membership from different perspectives which have not been previously examined. With this in mind, this study seeks to establish the relationship between low coverage of the NHIS and clients’ occasional unawareness of their membership status. The study further examines the factors associated with being unaware of one’s insurance coverage status.

This study is relevant to understanding the factors militating against active coverage on the NHIS in Ghana. The findings of this study contribute to building the evidence on areas of focus towards improving coverage of Ghana’s NHIS. Such evidence is also critically needed in order to advocate and pursue interventions in the context of other low- and middle-income countries pursuing universal health coverage through social insurance programs.

### Theoretical underpinnings of the study

It is often assumed that motivations that induce individuals to subscribe to health innovations including insurance programs will sustain adherence. However, adherence has been shown to be predicated on several factors including the ability to sustain premiums payments, level of understanding of how insurance operates, personal circumstances and motivation among others [22, 28, 29]. Indeed, Edwards Administrative Influence Model posits that communication, resources, disposition, and bureaucratic structure are key variables to the success of any policy initiatives [30]. Sabatier and Mazmanian bottom-up and top-down implementation model framework (1989) alludes to the fact that policy-making is a continuous process of formulation, implementation, and reformulation [31]. This suggests that beyond the initial process of enrolling individuals into insurance programs, there must be consistent and continuous efforts in ensuring adherence. Initial enthusiasm following program initiations often begin to falter and thus requires continuous appraisals and reorganization. Ghana’s NHIS program is predicated on continuous annual renewal of premiums. What this means is that merely holding of the card provided at initial enrollment does not necessarily entitle such individuals to continuous service. Unfortunately, in settings where the level of education is still low, there is a possibility that individuals may be holding cards which have expired without necessarily being aware that such registration has lapsed. By contributing evidence on the key drivers of low active enrollment, this study seeks to inform the process of policy reform of the NHIS in Ghana.

## Methods

### Study setting

The data used in this study were collected in the Upper East Region (UER), a locality in north-eastern Ghana with a population of slightly over one million in a land area of about 8842sq km, comprising 2.7% of the land mass of the country [2, 32]. The UER environment is primarily arid savanna grassland with a climate comprised of an annual May to September rainy season. About 70% of all UER residents are engaged in subsistence rain-fed agriculture. However, over-cropping and increasingly erratic rainfall have diminished agricultural productivity, exacerbating pervasive poverty and accelerating migration to the cities of southern Ghana [33]. As a consequence of these circumstances, the UER ranks among Ghana’s three most impoverished regions with a poverty prevalence of 55% and 40% of the population have no formal education [34]. Total fertility rate (TFR) in the UER is 4.9 [35]. Given the development challenges represented by the UER context, the region is typical of many rural and semi-urban communities in Sahelian Africa. Research findings from the UER are potentially relevant to many rural communities in sub-Saharan Africa.

Although the UER NHIS enrollment rate is 60%, the prevalence of actual coverage is only 40% [2] because enrollees often fail to comply with annual enrollment requirements. The UER was chosen for this study because this continuity of coverage problem can be researched in the region, owing to its long history of research on social and economic correlates of health behavior and also because the UER has pioneered health system development policies for health-deprived areas of Ghana [36, 37].

### Data source and sampling strategy

A Ghana Health Service sponsored health systems strengthening project was conducted in three districts of the UER over the 2010 to 2015 period that was known as the Ghana Essential Health Services Program (GEHIP). Four UER districts served as comparison areas, Details of the GEHIP program are described elsewhere [36, 38]. The terminal survey of GEHIP collected data on various health indicators including information on enrollment into the NHIS and characteristics of the UER population that could explain variance in awareness of compliance with renewal requirements. The GEHIP terminal survey was a cross-sectional study of 5914 women aged from 15 to 49 years. A two-stage sampling approach was used in the data collection process. First of all, the Ghana Statistical Service (GSS) sampled and provided 66 clusters comprised of census enumeration areas dispersed across the region. Guided by this sampling frame, physical identification of each cluster was done followed by a listing of members of all households located in each sample cluster. The second stage of sampling then involved the sampling of households proportional to the population size. Within sampled households, all resident women from age 15 to 49 years were eligible to be interviewed. During this survey, two questions related to NHIS were asked: “have you ever registered with the NHIS?” and “do you currently have a valid NHIS card?” If the answer to the second question is yes, the interviewer requested respondents to produce their card. Interviewer review of dates recorded on cards provided confirmation of coverage validity.

This analysis relies on these two questions, and dates that are validated, to explore the prevalence of respondents who are “unaware” that their NHIS coverage had expired. This information was analyzed in conjunction with survey respondent characteristics which permits appraisal of the covariates of unawareness of insurance coverage status among women who believe that they were insured by the NHIS at the time of the survey.

### Data analysis

STATA 14 software was used for the analysis; basic descriptive statistics involving numbers and percentages are used to describe the composition of variables, and cross-tabulations involving chi-square test of association is employed in bivariate analysis to identify variables that are associated with respondents who do not know the validity of their NHIS cards. Multivariate analyses apply binary logistic regression to the estimation of determinants of unawareness of the invalidity of one’s NHIS card. The main outcome of interest is “unawareness of card validity status” among respondents who claim to be covered by NHIS insurance. The variables that have been tested in the bivariate analysis are age group, educational level, functional literacy (ability to read and write), area of residence, marital status, household socio-economic status (wealth index), religion, occupation, and women autonomy. Only variables that were significant in the bivariate analysis were included in the multivariate regression model.

The variable for household socio-economic status (wealth index) and women autonomy was generated using principal component analysis (PCA). In the case of wealth index, household assets were used as a proxy for wealth; PCA is an approach that involves the use of scoring factors of each asset used to form an index for each household [39, 40]. For the autonomy variable, the six questions are as follows: Who usually takes decisions about major household purchases? Can you visit a friend or relative without permission? Who decides how money earned is spent in this household? Can you refuse to have sex with your husband without any severe consequences? Who makes decisions about purchases for daily needs? And do you need permission to seek care at a health facility? The answers to these questions were put together using PCA to generate the variable “autonomy” with three categories (autonomous, semi-autonomous, and not autonomous).

All other variables were recoded to correspond with available standard formats in the literature. The independent variables presented above were tested for multicollinearity using the variance inflation factor (VIF) prior to the logistic regression analysis, the VIF was 2.08 signifying no *multicollinearity* between variables (a VIF value > 5 would have signified high multicollinearity). We also applied sample weighting in our regressing analysis to ensure that findings are representative of the study area and the population. In both bivariate and multivariate analysis, a *p* value less than or equal to 0.05 is regarded as significant association while *p* values above 0.05 are deemed not significant. Both *p* values and confidence intervals have been reported in the regression model.

## Results

### Background characteristics of study respondents

**Fig. 1:**
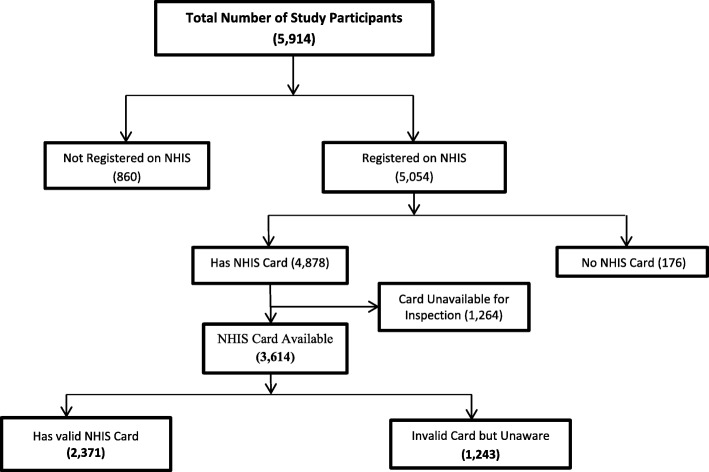
Flow chart showing unawareness of NHIS card validity status

Out of the total 5914 respondents, 5054 representing about 85.5% were registered with the national health insurance scheme. Out of these respondents, 4878 claimed that they had valid NHIS cards but only 3614 had their NHIS cards available for inspection. Of the respondents who had available cards, 1243 (34.5%) had expired cards. Since these respondents had claimed that they were insured, they constitute a sample of women who were unaware of the expiration of their insurance coverage (Fig. 1).

**Fig. 2:**
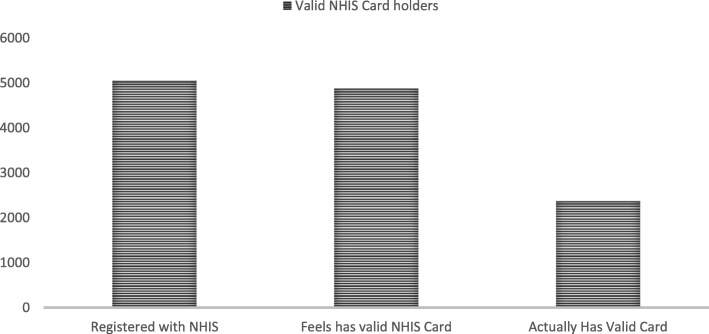
NHIS card holders

Figure 2 shows respondents that are registered with the NHIS, those who feel they have valid cards and those who actually have a valid card upon inspection by the data collectors.

Table 1 shows the background characteristics of the 3614 respondents included in this analysis. Over half (58.5%) had no formal education and over two thirds (68.9%) were illiterate. A majority of the respondents were currently married (70.4%), although nearly a quarter were single (24.0%) while a small percentage were widowed (4.8%), divorced, or separated (0.8%). Of those who were currently married, a third (34.8%) were in polygamous unions. A plurality of the respondents were farmers (34.1%), while 18.9% were students, with the remainder comprised of women with no occupation. Over half of the respondents professed Christianity as their household religion (56.5%), about a third were Muslim 33.1% and only 9.0% were practitioners of traditional religion. Only rarely did respondents claim to have no religion (1.4%). Three quarters of the respondents (76.8%) were resident in rural settings while only 9.0% were resident in urban settings. The rest (14.2%) were resident in semi-urban settings. When it comes to household socio-economic status, quintile 2 (the poorer category) was the highest (33.5%), while quintile 3 was the least (7.3%). As per our women-autonomy variable, 34.0% of married respondents were not autonomous while about 19.6% were autonomous, the rest were semi-autonomous.

**Table 1:** Background characteristics of respondents (*n* = 36140)

Background characteristics	Number	Percent
Age group		
15–19	760	21.03
20–34	1621	44.85
35–49	1233	34.12
Highest level of education		
None	2113	58.47
Primary/junior high school	1201	33.23
Secondary/tertiary	283	7.83
Other	17	0.47
Functional literacy (ability to read and understand)		
Yes	1123	31.07
No	2491	68.93
Marital status		
Not married yet (single)	866	23.96
Married	2544	70.39
Widowed	173	4.79
Devoiced/separated	28	0.77
Marriage type		
Polygamous	885	34.79
Monogamous	1639	64.43
Do not know	20	0.79
Occupation		
Farming	1231	34.06
Trading	602	16.66
Artisan	450	12.45
No occupation/housewife	483	13.36
Civil servant	52	1.44
Student	683	18.90
Other	113	3.13
Religion		
Christianity	2040	56.45
Traditional	326	9.02
Islam	1196	33.09
No religion	52	1.44
Area of residence		
Urban	326	9.02
Semi-urban	512	14.17
Rural	2776	76.81
District of residence		
Bolga M.	346	9.62
Bongo	472	13.13
Builsa	590	16.41
Garu/Tempani	949	26.40
Bawku West	322	8.96
Talensi/Nabdam	352	9.79
Bawku East	564	15.69
SES (wealth index)		
Quintile 1 (poorest)	760	21.03
Quintile 2	1212	33.54
Quintile 3	263	7.28
Quintile 4	727	20.12
Quintile 5 (richest)	652	18.04
Autonomy		
Not autonomous	1230	34.03
Semi-autonomous	607	16.80
Autonomous	707	19.56

### Bivariate analysis

Of all the variables that were analyzed using the chi-square test of association, level of education, functional literacy, occupation, religion, area of residence, district of residence, and socio-economic status were significantly associated with unawareness of NHIS card validity in bivariate analysis. Table 2 provides details of this analysis. Other important variables that did not show any significant association with our dependent variable are age, marital status, marriage type, and autonomy.

**Table 2:** Bivariate analysis of unawareness of active NHIS enrollment status (*χ*^2^ test)

Determinants	Active enrollment status		
	Seen and valid, *n* (%)	Seen and invalid, *n* (%)	*p* value
Age group			
15–19	513 (67)	247 (33)	
20–34	1064 (66)	557 (34)	0.366
35–49	794 (64)	439 (36)	
Level of education			
None	1366 (65)	747 (35)	
Primary/junior high school	786 (64)	415 (36)	0.034
Secondary/tertiary	208 (73)	75 (27)	
Other	11 (65)	6 (35)	
Functional literacy (ability to read and understand)			
Yes	768 (68)	355 (32)	0.018
No	1603 (64)	888 (36)	
Marital status			
Not married yet (single)	583 (67)	283 (33)	
Married	1656 (65)	888 (35)	0.65
Widowed	113 (64)	63 (36)	
Divorced/separated	19 (68)	9 (32)	
Marriage type			
Polygamous	561 (63)	324 (37)	0.358
Monogamous	1083 (66)	556 (34)	
Do not know	12 (60)	8 (40)	
Occupation			
Farming	757 (61)	474 (39)	
Trading	403 (67)	199 (33)	
Artisan	317 (70)	133 (30)	
No occupation/housewife	321 (66)	162 (34)	0.003
Civil servant	41 (79)	11 (21)	
Student	453 (66)	230 (34)	
Other	79 (70)	34 (30)	
Religion			
Christianity	1313 (64)	727 (36)	
Traditional religion	200 (61)	126 (39)	< 0.001
Islam	834 (70)	362 (30)	
No religion	24 (46)	28 (54)	
Area of residence			
Urban	216 (66)	110 (34)	0.613
Semi-urban	345 (67)	167 (33)	
Rural	1810 (65)	966 (35)	
District of residence			
Bolga M.	211 (61)	135 (39)	
Bongo	298 (63)	174 (37)	
Builsa	374 (63)	216 (37)	
Garu/Tempani	651 (69)	298 (31)	< 0.001
Bawku West	228 (71)	94 (29)	
Talensi/Nabdam	207 (59)	146 (41)	
Bawku East	391 (69)	173 (31)	
SES (wealth index)			
Quintile 1 (poorest)	459 (60)	301 (40)	
Quintile 2	788 (65)	424 (35)	< 0.001
Quintile 3	167 (63)	96 (37)	
Quintile 4	494 (68)	233 (32)	
Quintile 5 (richest)	463 (71)	189 (29)	
Autonomy			
Not autonomous	820 (67)	410 (33)	0.234
Semi-autonomous	391 (64)	216 (36)	
Autonomous	445 (63)	262 (37)	

Figures in parentheses are percentages

### Multivariate analysis

Neither level of education nor functional literacy was significant in the multivariate regression analysis. Occupational status was significant; all other occupation categories were less likely to be unaware of their NHIS status compared to farming, and this was significant for all occupations except students and civil servants. Thus, farmers were those more likely to be unaware of their card validity. Traders, artisans, housewives, and others are 24%, 35%, 25%, and 40% less likely to be unaware of their card validity compared to farmers.

Respondents who said they are not affiliated to any of the three religions were two times more likely to be unaware of their card invalidity compared to those affiliated to Christianity. District of residence was significantly associated with unawareness of NHIS card validity. Compared to Bolgatanga, the regional capital, being resident in Garu-Tempani, Bawku West, and Bawku East was significantly more protective against unawareness of NHIS card validity.

The socio-economic (wealth index) variable was the most interesting to observe. It can be observed clearly that increasing wealth index meant more protection from the tendency of unawareness. Quintile 4 and quintile 5 (richest category) were 21% and 27%, respectively, more protective and significantly associated with unawareness compared with the poorest category. Table 3 presents the details of the multivariate analysis.

**Table 3:** Multivariate analysis of unawareness of NHIS status of respondents; logistic regression model

	Odds ratio	*P* > *z*	95% Conf. interval	
Level of education (compared with no education)				
Primary/junior high sch.	1.09	0.454	0.87	1.38
Secondary/tertiary	0.82	0.332	0.54	1.23
Other	0.81	0.698	0.27	2.40
Functional literacy (compared with yes)				
No	1.20	0.208	0.90	1.59
Occupation (compared with farming)				
Trading	0.76	0.018	0.61	0.96
Artisan	0.65	0.001	0.50	0.84
No occupation/housewife	0.75	0.021	0.59	0.96
Civil servant	0.52	0.093	0.24	1.11
Student	0.93	0.619	0.69	1.25
Other	0.60	0.025	0.39	0.94
Religion (compared with Christianity)				
Traditional religion	0.93	0.588	0.71	1.21
Islam	0.94	0.515	0.77	1.14
No religion	2.04	0.016	1.14	3.64
Area of residence (compared with urban)				
Semi-urban	0.94	0.697	0.67	1.30
Rural	0.97	0.849	0.73	1.30
District of residence (compared with Bolgatanga)				
Bongo	0.81	0.167	0.59	1.10
Builsa	0.78	0.116	0.58	1.06
Garu/Tempani	0.59	< 0.001	0.44	0.79
Bawku West	0.58	0.001	0.41	0.81
Talensi/Nabdam	0.99	0.934	0.72	1.36
Bawku East	0.61	0.003	0.44	0.85
SES (wealth index) (compared with Q1 poorest)				
Quintile 2	0.85	0.111	0.70	1.04
Quintile 3	0.94	0.676	0.69	1.27
Quintile 4	0.79	0.045	0.63	0.99
Quintile 5 (richest)	0.73	0.015	0.57	0.94

## Discussion

This study has explored the possibility that being unaware of one’s health insurance status is a major contributor to the persistent low active enrollment of Ghana’s national health insurance scheme. Findings of the study clearly demonstrate that as high as 34.5% of respondents who felt that they had active insurance cards were indeed possessing invalid cards. This figure represents only those whose cards were available for inspection by the data collectors. About 1264 respondents representing 25.9% of respondents who said they had valid cards could not produce them for inspection; this implies the proportion of respondents that are unaware of the true status of their insurance cards could be more than 34.5%.

Given that as high as 85.5% of respondents have been registered with the scheme but less than half of that number could produce valid insurance cards while 34.5% felt they were insured but turn out not to be insured clearly demonstrates that a substantial number of insurance holders are often unaware of the expiration of their cards in order to have them renewed.

As stated earlier, the trauma and consequences of going to a health facility to seek care only to be told at the point of need that the insurance card the person is holding has expired could be devastating, especially to the poor and vulnerable. With these experiences, such individuals would either be careful next time to always check their insurance cards or may decide not to register due to the anger and resentment. Indeed, a previous study has shown that key among the determinants of enrollment are poor social infrastructure, vulnerability among social groups, and weak NHIS systems [41].

The implication of this high phenomenon of unawareness of NHIS card expiration on Ghana’s progress towards universal health coverage is enormous. The national health insurance has been positioned as one of the key pillars for ensuring universal access to affordable and equitable healthcare in Ghana; however, persistently low active enrollment is a threat to the operations, sustainability, and impact of the NHIS in removing financial barriers to healthcare [25].

In examining the determinants predicting unawareness of card validity, respondents’ occupation, district of residence, and socio-economic status were significantly associated with this phenomenon while factors such as age, level of education, functional literacy, marital status, area of residence, and autonomy were not significantly associated with our dependent variable.

Age of respondent was not significant in the bivariate analysis which is in congruence with previous studies [2, 29], so it was not included in the regression model.

Both the level of education and functional literacy were significant in bivariate analysis. However, in the multivariate analysis, the significance of these two important variables got completely lost. Some previous studies have identified education to be a significant predictor of enrollment [29, 42].

For example, Alatinga and Williams in their exploration of determinants of household enrollment found that households whose heads were educated had greater chances of being enrolled than families with uneducated household heads [28]. Also, a study by Akazili et al. found that educated women are more likely to be insured than uneducated ones [2]. Other studies that examined factors influencing enrollment onto Ghana’s NHIS also confirm education to be influential in determining enrollment [28, 29, 43]. Therefore, inferring from previous studies and results from this present study, it is highly plausible that while educational status influences enrollment, it is immaterial in influencing the chances that one would be unaware of one’s insurance card validity. And that irrespective of one’s educational status, everyone is equally liable to not knowing the validity of his/her insurance card.

Marital status and marriage type were not significant in bivariate analysis and were therefore not included in the multivariate regression analysis. This suggests that marital status is not a predictor of unawareness of insurance card validity. Although some previous studies have documented that married people are more likely to be insured than non-married people [2, 29], this study has shown that marriage is not protective of unawareness of card validity; therefore, campaigns to improve awareness of card validity should not be selective of marital status.

Occupational status has emerged as one of the strong predictors of unawareness of insurance card expiration among the study participants. Women who are involved in farming are significantly more likely not to know the expiration status of their cards than any other occupation category. Alatinga and Williams have documented that people engaged in formal employment such as civil servants are more likely to be actively enrolled than those in the informal sector [28]. In Ghana, farming constitutes a large fraction of the informal sector workers, indeed 34.1% representing 1231 of respondents in this analysis are farmers; therefore, this situation is worrying particularly because incomes of farmers in Ghana are very meager and so they are more prone to catastrophic healthcare payments when sick. Immediate efforts would need to be put in place to ensure that people in this occupation category are made aware and supported to enroll and continue to maintain active enrollment year on year.

Respondents not affiliated with a religion were found to be two times more likely to be unaware of their NHIS card expiration status as compared to Christians. A previous study has shown that people affiliated with Christian religion are more likely to be enrolled onto the NHIS than those affiliated with traditional religion while those affiliated with Islamic religion are also more likely to enroll compared to those affiliated with Christian religion [2] socio-cultural and religious factors especially those that enforce male dominance have been found to contribute to low NHIS update and NHIS card renewal in some parts of Ghana [41]. Our study, however, seems to suggest no significant association between unawareness of card expiration status when comparing the three main religions in Ghana. Hence, the chance of being unaware of insurance card validity is nondiscriminatory of religious affiliation.

Area of residence, unexpectedly, was not significantly associated with unawareness of card expiration status in multivariate analysis although it was in bivariate analysis. Previous qualitative studies have identified low NHIS update in communities that lack health infrastructure and have poor means of transportation and limited communication infrastructure [41], In this current study, we however did not find unawareness of NHIS card expiration status to be associated with place of residence. This is most likely due to the widespread development of community-based health facilities in the Upper East region, where this study was conducted. The implication of our finding in this study setting is that being unaware of one’s insurance card expiration status is not peculiar to being resident in a rural, urban, or semi-urban setting but it cuts across all residential settings.

District of residence was, however, strongly associated with unawareness of insurance card expiration status. Residents of Garu-Tempani District, Bawku West, and Bawku East Districts were 41%, 42%, and 39%, less likely to be unaware of their insurance card expiration status compared to residents of Bolgatanga; the regional capital (*p* value < 0.05). Even residents of Bongo, Builsa, and Talensi/Nabdam Districts were 19%, 22%, and 1% less likely to be unaware of their card validity compared to Bolgatanga; however, this was not significant (*p* value > 0.05). It can thus be inferred that residents of Bolgantanga, the regional capital, are more likely to be unaware of their card expiration status than residents in most parts of the region. Inferring from previous literature [41], the relative difference in unawareness of card expiration status across districts in the region may be attributed to differences in health infrastructure and effectiveness of NHIS workers in the various districts.

With regard to socio-economic status, compared to quintile 1 (poorest), quintile 5 (richest) and quintile 4 were 27% and 21% less likely to be unaware of their card expiration status. Quintile 3 and 2 were also 6% and 15% less likely to be unaware of card expiration status compared to quintile 1 (poorest). The results are particularly significant for quintile 5 (richest) and quintile 4. There was a consistent decrease in the likelihood of unawareness of health insurance card expiration status with increasing socio-economic status. Virtually all studies that have examined determinants of enrollment onto the national health insurance scheme have documented low socio-economic status to be significantly associated with non-enrollment [2, 28, 29, 41]. Inferring from results of this study, one can argue that the poor are unable to enroll and maintain an active membership in the insurance program not only because of an inability to pay as identified by most studies [41], but they may be busily engaged in survival-related activities so much to the extent that they are unable to keep check of the validity or otherwise of their insurance cards. Efforts to address this problem of unawares would have to target the poor and provide them with the needed support.

### Study limitations

This study was conducted on a predominantly rural population and focused on only women so findings may not be generalizable to the whole country. Also, it was not possible to check the NHIS cards of all study participants since others did not have theirs available for inspection. Furthermore, as a quantitative cross-sectional study, this study is limited in its ability to provide an in-depth understanding of why respondents are unaware of their insurance card expiration status. This notwithstanding, this study has unearthed an important contributor to the low active enrollment of Ghana’s national health insurance scheme.

## Conclusion

This study has demonstrated that unawareness of card expiration status is a contributor to low active membership of the NHIS. Efforts to advance Ghana’s goal of achieving universal health coverage through the national health insurance scheme must address the issue of unawareness of insurance card expiration. The study shows that being unaware of one’s card expiration is irrespective of age, education, marital status, religion, or area of residence (rural/urban). For the NHIS to play its fundamental role towards the achievement of universal health coverage in Ghana, its operations and policies need to be revised to include mechanisms (including educational campaigns) for reminding members whose NHIS cards are due to renewals. Targeted efforts are required to ensure that people of low socio-economic status and farmers are made aware and supported to enroll and continue to remain active enrollment.

## Data Availability

The dataset used in this study is available upon request to the corresponding author of this paper.

## Abbreviations

GEHIP: Ghana Essential Health Intervention Project
GSS: Ghana Statistical Service
NHIS: National Health Insurance Scheme
SAP: Structural adjustment programs
TFR: Total fertility rate
